# A2 UVB ON SKIN EXPOSURE MODULATES INTESTINAL HOMEOSTASIS IN MICE

**DOI:** 10.1093/jcag/gwad061.002

**Published:** 2024-02-14

**Authors:** M C Jimenez Sanchez, H Yang, J Dutz, H Sham, B Vallance

**Affiliations:** Department of Medicine, The University of British Columbia, Vancouver, BC, Canada; Department of Medicine, The University of British Columbia, Vancouver, BC, Canada; Department of Medicine, The University of British Columbia, Vancouver, BC, Canada; Department of Medicine, The University of British Columbia, Vancouver, BC, Canada; Department of Medicine, The University of British Columbia, Vancouver, BC, Canada

## Abstract

**Background:**

Inflammatory diseases, like IBD, are on the rise in Western societies, in concert with changes in the environment, such as reduced sun exposure. Ultraviolet light (UV) B's effects on the skin include upregulating vitamin D and Aryl hydrocarbon receptor (Ahr) signalling, but its potential influence on distant sites, like the gut, are not well-studied. The gut and skin share a strong link, with some GI patients also experiencing skin diseases (O’Neill et al., 2016). We sought to understand how UVB exposure affects gut health, gut microbiota and its metabolic by-products. We hypothesize that UVB will have a significant impact on gut health.

**Aims:**

Test the effect of UVB light using vitamin D deficient (-) and sufficient (+) diets.

Elucidate Ahr activation in the small intestine and colon after UVB exposure.

Explore the gut metabolome after UVB exposure.

**Methods:**

Female C57BL/6 mice were fed vit D (+) or (-) diets, anaesthetized, and then 8 cm^2^ of their dorsal skin was shaved and exposed to UVB (250 mJ/cm^2^, 234 sec, 311 nm). One single dose (sample collection after 4 or 10 h) or chronic dosage (9 days every other day). Mice were euthanized to collect serum, cecal content, and stool samples (HILIC negative profile and 16S sequencing). EROD assay was used to test Ahr activation of CYP1A1.

**Results:**

UVB exposure did not cause skin inflammation, but instead induced photoadaptation. When mice with vit D (-) diets were exposed to UVB, their vit D levels rose to match those on vit D (+) diets. Intestinal luminal contents induced a significant 3X increase in Ahr activation at 10 h post-exposure. After chronic UVB exposure, Ahr/CYP1A1 activity increased in Peyer’s patches (p=0.0012) and colon (p=0.01). Serum levels of AhR agonist metabolites post-UVB exposure showed significant increases in kynurenine and indole-3-acetic acid (IAA) at 10 h, while in the feces, Tryptophan levels decreased (p=0.042), but IAA significantly increased (p=0.006). IAA is a known Ahr ligand and produced by gut microbiota when they metabolize Tryptophan, indicating a microbiota response to UVB skin exposure. This was accompanied by an increase in the short chain fatty acids (butyrate, propionate, acetic acid) as well as other metabolites, despite no overt changes in microbiota composition. After 9 days of UVB, significantly increased numbers of goblet cells/crypt in both the ileum and colon.

**Conclusions:**

Our study demonstrates that UVB exposure on the skin leads to increased vitamin D serum levels and Ahr activation along the gastrointestinal tract. It also induces potentially beneficial metabolic changes in the gut microbiota and differentiation of the gut epithelium, highlighting sun exposure's potential intestinal health benefits.

**Funding Agencies:**

CCC, CIHR, None

